# Synthetic MRI, multiplexed sensitivity encoding, and BI-RADS for benign and malignant breast cancer discrimination

**DOI:** 10.3389/fonc.2022.1080580

**Published:** 2023-02-03

**Authors:** Jinrui Liu, Mengying Xu, Jialiang Ren, Zhihao Li, Lu Xi, Bing Chen

**Affiliations:** ^1^ School of Clinical Medicine, Ningxia Medical University, Yinchuan, China; ^2^ Department of Radiology, General Hospital of Ningxia Medical University, Yinchuan, China; ^3^ Department of Pharmaceuticals Diagnostics, GE Healthcare, Beijing, China; ^4^ Department of Pharmaceuticals Diagnostics, GE Healthcare, Xi’an, China; ^5^ Sales Department, GE Healthcare, Yinchuan, China

**Keywords:** breast cancer, synthetic magnetic resonance imaging (syMRI), diffusion-weighted imaging, multiplexed sensitivity-encoding, nomogram

## Abstract

**Objective:**

To assess the diagnostic value of predictive models based on synthetic magnetic resonance imaging (syMRI), multiplexed sensitivity encoding (MUSE) sequences, and Breast Imaging Reporting and Data System (BI-RADS) in the differentiation of benign and malignant breast lesions.

**Methods:**

Clinical and MRI data of 158 patients with breast lesions who underwent dynamic contrast-enhanced MRI (DCE-MRI), syMRI, and MUSE sequences between September 2019 and December 2020 were retrospectively collected. The apparent diffusion coefficient (ADC) values of MUSE and quantitative relaxation parameters (longitudinal and transverse relaxation times [T1, T2], and proton density [PD] values) of syMRI were measured, and the parameter variation values and change in their ratios were calculated. The patients were randomly divided into training (n = 111) and validation (n = 47) groups at a ratio of 7:3. A nomogram was built based on univariate and multivariate logistic regression analyses in the training group and was verified in the validation group. The discriminatory and predictive capacities of the nomogram were assessed by the receiver operating characteristic curve and area under the curve (AUC). The AUC was compared by DeLong test.

**Results:**

In the training group, univariate analysis showed that age, lesion diameter, menopausal status, ADC, T2_pre_, PD_pre_, PD_Gd_, T2_Delta_, and T2_ratio_ were significantly different between benign and malignant breast lesions (*P* < 0.05). Multivariate logistic regression analysis showed that ADC and T2_pre_ were significant variables (all *P* < 0.05) in breast cancer diagnosis. The quantitative model (model A: ADC, T2_pre_), BI-RADS model (model B), and multi-parameter model (model C: ADC, T2_pre_, BI-RADS) were established by combining the above independent variables, among which model C had the highest diagnostic performance, with AUC of 0.965 and 0.986 in the training and validation groups, respectively.

**Conclusions:**

The prediction model established based on syMRI, MUSE sequence, and BI-RADS is helpful for clinical differentiation of breast tumors and provides more accurate information for individualized diagnosis.

## 1 Introduction

Breast cancer has become the most commonly diagnosed cancer, seriously threatening the health of women (1). The early detection and diagnosis of breast diseases are crucial for the prognosis of breast cancer. Dynamic contrast-enhanced magnetic resonance imaging (DCE-MRI) has been widely applied in the differential diagnosis of breast diseases (2–5). The Breast Imaging Reporting and Data System (BI-RADS) is a standardized acquisition and interpretation system for breast MRI. Breast MRI typically classify lesions based on the BI-RADS criteria. Although high accuracy has been reported, the specificity of the BI-RADS diagnosis varies widely (6–8). In addition, the BI-RADS classification is related to the experience of the radiologist and there is no definite diagnosis of the lesion.

Diffusion weighted imaging (DWI) is an effective MRI technique that can noninvasively measure the diffusion of water molecules in tissue (9). DWI can provide information on lesions at the cellular and molecular levels, and is an effective parameter for distinguishing benign and malignant lesions (5). However, DWI is mainly based on single-shot echo-planar imaging (SS-EPI), which is prone to geometric distortion (10). Multiplexed sensitivity encoding (MUSE) DWI integrates a sensitivity-encoding parallel imaging method and achieves a better signal-to-noise ratio (SNR) due to its improved matrix inversion conditioning (11, 12). MUSE-DWI can acquire high-spatial-resolution images within a clinically feasible acquisition time and reduce ghosting artifacts and geometric distortions (11). MUSE-DWI sequence has been applied in brain (10) and breast (11) and has shown higher image quality than traditional SS-EPI.

Recently, a multi-contrast and one-stop relaxation quantitative technique called synthetic MRI (syMRI) has emerged, which can simultaneously quantify tissues’ synthetic relaxometry (longitudinal and transverse relaxation times [T1, T2]) and proton density (PD), as well as a variety of weighted images (13, 14). Tissue relaxation times form the fundamental basis of soft tissue contrast and anatomical imaging with MRI (15). As the malignancy of the tumor increases, the change in relaxation time can be measured by MRI (16). This technique has been successfully applied for the brain (16, 17), breast (18–21), and prostate (22) and has shown good diagnostic performance. However, conclusions on breast diagnosis are still inconsistent, and comparisons between MUSE-DWI and BI-RADS are rarely performed.

A nomogram is a graphic calculating scale tool that provides a predictive model for individual prognosis (23). By quantifying independent risk factors, the total score of the nomogram corresponds to the risk prediction value, which can succinctly and intuitively reflect the personalized prediction. This study used syMRI combined with the MUSE sequence to analyze its diagnostic performance for breast lesions. In addition, combined with the BI-RADS, a nomogram was established to explore its diagnostic performance in benign and malignant breast lesions.

## 2 Materials and methods

### 2.1 Patients

**Figure 1:**
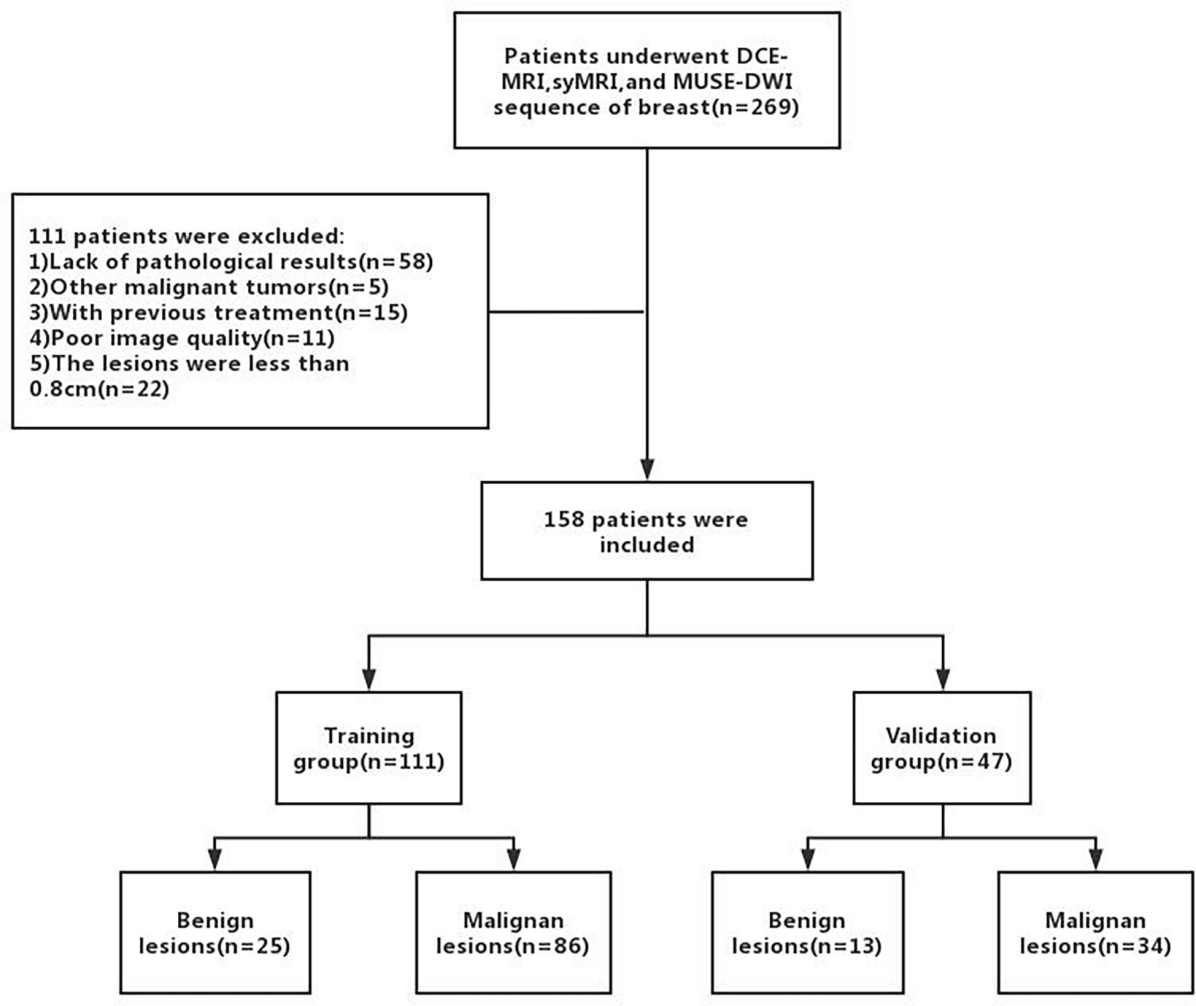
The flowchart of patient enrollment.

A total of 158 female patients (mean age, 50.19 ± 11.81 years; age range, 22-80 years; 38 with benign lesions and 120 with malignant lesions) who satisfied the inclusion criteria were enrolled between September 2019 and December 2020 (**Figure 1**). The inclusion criteria were as follows (1): Patients who underwent breast DCE-MRI, syMRI, and MUSE-DWI sequences, and the same syMRI sequence parameters were used for scanning before and after enhancement; 2) Surgery or needle biopsy performed within two weeks of MRI; 3) No surgery, chemotherapy, radiotherapy, or other related treatments before MRI examination; 4) Lesion diameter > 0.8 cm; 5) Patients with sufficient MRI image quality for quantitative measurement. All patients were randomly divided into the training group (111 cases) and validation group (47 cases) at a ratio of 7:3. The training group included 25 benign and 86 malignant patients, and the validation group included 13 benign and 34 malignant patients (**Table 1**). This study was approved by the Institutional Ethics Board of the hospital (KYLL-2022-0551), and informed consents were waived.

**Table 1:** Basic characteristics of the study population.

Variables	Training group		Validation group	
	Benign (N = 25)	Malignant (N = 86)	Benign (N = 13)	Malignant (N = 34)
Age (year)	40.64 ± 11.39	53.73 ± 10.06	38.31 ± 7.83	52.79 ± 11.06
Diameter (cm)	2.70 (1.75, 5.05)	2.20 (1.50, 2.90)	1.80 (1.20, 2.50)	2.10 (1.68, 2.80)
Menopausal state, n (%)				
Post	3 (12.00%)	48 (55.80%)	1 (7.70%)	19 (55.90%)
Pre	22 (88.00%)	38 (44.20%)	12 (92.30%)	15 (44.10%)
Family history, n (%)				
No	25 (100.00%)	83 (96.50%)	13 (100.00%)	33 (97.10%)
Yes	0 (0.00%)	3 (3.50%)	0 (0.00%)	1 (2.90%)
CA125, n (%)				
Negative	25 (100.00%)	85 (98.80%)	13 (100.00%)	32 (94.10%)
Positive	0 (0.00%)	1 (1.20%)	0 (0.00%)	2 (5.9%)
CA153, n (%)				
Negative	25 (100.00%)	82 (95.30%)	13 (100.00%)	34 (100.00%)
Positive	0 (0.00%)	4 (4.70%)	0 (0.00%)	0 (0.00%)
BI-RADS, n (%)				
3, 4a	16 (64.00%)	2 (2.3%)	10 (76.90%)	1 (2.90%)
4b, 4c, 5	9 (36.00%)	84 (97.70%)	3 (23.10%)	33 (97.10%)
ADC (×10^−3^ mm^2^/s)	1.31 (1.16, 1.50)	0.99 (0.92, 1.07)	1.52 (1.30, 1.66)	0.97 (0.89, 1.13)
T1_pre_ (msec)	1249.33 (1107.83, 1459.33)	1266.83 (1154.25, 1355.50)	1296 (1186.83, 1644.83)	1236.83 (1130.92, 1318.17)
T2_pre_ (msec)	87.30 ± 15.05	73.28 ± 1.00	90.69 ± 17.19	73.24 ± 9.15
PD_pre_ (pu)	73.43 (57.63, 82.83)	58.13 (52.59, 66.58)	63.2 (54.88, 70.55)	58.70 (51.81, 64.96)
T1_Gd_(msec)	481.67 (438.17, 523.17)	519.83 (474.00, 568.17)	496.67 (414.00, 633.17)	504.33 (466.67, 558.50)
T2_Gd_ (msec)	70.00 (68.17, 76.50)	65.50 (58.25, 70.83)	72.67 (63.5, 78.33)	63.50 (57.00, 67.75)
PD_Gd_ (pu)	83.66 ± 14.88	74.78 ± 13.44	64.63 ± 10.74	59.80 ± 12.38
T1_Delta_ (msec)	-780.67 (-1003.83, -610.00)	-731.33 (-857.50, -637.92)	-849.67 (-1080, -654.17)	-690.33 (-823.42, -615.83)
T2_Delta_ (msec)	-17.13 (-20.50, -12.00)	-9.17 (-14.42, -4.83)	-19.67 (-33.17, -9.50)	-10.50 (-14.75, -4.25)
PD_Delta_ (pu)	14.17 ± 8.97	14.24 ± 9.55	12.12 ± 7.95	14.57 ± 7.67
T1_ratio_ (msec)	-0.63 (-0.68, -0.56)	-0.59 (-0.64, -0.55)	-0.63 (-0.70, -0.55)	-0.59 (-0.64, -0.54)
T2_ratio_ (msec)	-0.19 (-0.23, -0.15)	-0.13 (-0.19, -0.07)	-0.21 (-0.33, -0.12)	-0.15 (-0.19, -0.06)
PD_ratio_ (pu)	0.22 ± 0.16	0.25 ± 0.17	0.19 ± 0.13	0.26 ± 0.14

### 2.2 MRI protocols

All patients underwent MR examinations using a 3.0 T whole-body scanner (Signa Architect, GE Healthcare, Milwaukee, Wisconsin, USA) with an eight-channel phased-array breast surface coil. All patients were scanned in the prone position and conventional MRI (including T1WI and T2WI) was performed first, followed by MUSE DWI and DCE-MRI sequences. Axial syMRI sequences (OAx MAGiC) were obtained before and after contrast injection, with consistent scan parameters. A rapid bolus of gadodiamide contrast agent (GE Healthcare, Ireland) was injected intravenously at a dose of 0.2 mL/kg with an injection rate of 2.5 mL/s, followed by a flush of 20 mL normal saline at a rate of 3 mL/s. **Table 2** lists the detailed parameters of the imaging sequences.

**Table 2:** Imaging protocols for MRI.

Parameter	T_1_WI	T_2_WI	DWI	DCE-MRI	SyMRI
Sequence	FSE	FLEX	MUSE	DISCO+C	MAGiC
Orientation	Ax	Ax	Ax	Ax	Ax
Fat suppression	No	Yes	Yes	Yes	No
Repetition time (msec)	626	4258.0	5000	3.8	4000
Echo time (msec)	Min Full	85	Minimum	Minimum	18.1, 90.5
Section thickness (mm)	5	5	5	1	5
No. of sections	28	28	20	170	24
b values (sec/mm^2^)	N/A	N/A	0/800	N/A	N/A
Field of view (cm)	32 × 32	32 × 32	30 × 15	32 × 32	32 × 32
Matrix	384 × 300	320 × 288	180 × 92	320 × 320	320 × 256
Bandwidth (Hz/pixel)	62.5	83.33	250	83.33	31.25
Acceleration factor	2	2	1	1	2
Scan time (min)	01:14	02:10	02:45	08:25	05:07

Ax, axial view; N/A, not available; FSE, fast spin-echo; MAGiC, magnetic resonance image compilation.

### 2.3 Image analysis

Two radiologists (R.J.L. and Y.M.X. with three and six years of experience in breast imaging, respectively), blinded to the pathology results, reviewed all the images with the dedicated Advantage Workstation (AW 4.7, GE Healthcare). Region-of-interests (ROIs) were manually drawn on the largest area of the lesion. All ROIs were placed from the solid portion of the lesion, excluding hemorrhagic necrosis or cystic lesions. The names of imaging parameter in **Table 3** follow the rule that prefixes T1, T2, and PD represent the quantitative relaxation indices, and suffixes pre, Gd, delta, and ratio represent before enhancement, after enhancement, difference between before and after enhancement, and ratios before and after enhancement, respectively, calculated as: ratio = (Gd-pre)/pre. In case of discrepancy in the opinions, the two observers negotiated and reached an agreement and then the final results were record. In addition, the BI-RADS categories of patients were extracted from radiology reports following the BI-RADS MRI protocol. According to clinical practice, BI-RADS 3 and BI-RADS 4a were classified as benign (i.e., malignancy could be excluded), and BI-RADS 4b, BI-RADS 4c, and BI-RADS 5 were classified as malignant (i.e., malignancy could not be excluded). This study compared the results of MRI diagnosis with pathological results to calculate the diagnostic efficacy.

**Table 3:** Diagnostic value of parameters in univariate and multivariate regression analysis in the training group.

Variables	Univariate logistic regression analysis				Multivariate logistic regression analysis(Variables with VIF >10 are removed)			
	2.50%	97.50%	OR	*P* value	2.50%	97.50%	OR	*P* value
Age	1.077	1.211	1.136	**< 0.001**	0.944	1.217	1.067	0.305
Diameter	0.483	0.853	0.660	**0.004**	0.447	1.410	0.808	0.458
Menopausal state	2.933	41.227	9.263	**< 0.001**	0.075	21.576	1.183	0.905
ADC	< 0.001	0.001	< 0.001	**< 0.001**	< 0.001	0.003	< 0.001	**< 0.001**
T1_pre_	0.997	1.002	1.000	0.687				
T2_pre_	0.831	0.936	0.888	**< 0.001**	0.819	0.978	0.906	**0.026**
PD_pre_	0.920	0.985	0.953	**0.005**	0.859	1.076	0.963	0.496
T1_Gd_	1.000	1.010	1.004	0.123				
T2_Gd_	0.939	1.008	0.973	0.128				
PD_Gd_	0.921	0.988	0.956	**0.012**	0.946	1.120	1.024	0.571
T1_Delta_	0.999	1.003	1.001	0.298				
T2_Delta_	1.050	1.213	1.119	**0.002**				
PD_Delta_	0.953	1.050	1.001	0.972				
T1_ratio_	0.173	14600.323	38.461	0.207				
T2_ratio_	6.626	425258.619	833.374	**0.017**				
PD_ratio_	0.209	44.378	2.911	0.430				

The meaning of the bold value is p<0.05.

### 2.4 Statistical analysis

All statistical analyses were performed using the R statistical software (The R Foundation; http://www.rproject.org;version4.1.0) and SPSS software (version 24.0; IBM Corp., Armonk, NY, USA). Continuous data were summarized as means ± standard deviation (M ± SD) or median and interquartile ranges (Q1, Q3) for normal or non-normal distribution data, as appropriate. The Kolmogorov-Smirnov test was used for normal distribution. Univariate logistic regression analysis was performed to identify indicators for diagnosing breast cancer. Variables with a p-value > 0.05 (in the univariate case) from the univariate logistic regression analysis were incorporated into multivariate regression analysis to identify independent factors. Based on the results of univariate and multivariate regression analysis, a nomogram was constructed using the “rms” package in R software. The diagnostic values of various predictive models were evaluated by the area under the curve (AUC) of the receiver operating characteristic (ROC) curve through the “riskRegression” R package. The DeLong test was used to evaluate differences in the AUC of each model. Accuracy, sensitivity, specificity, positive predictive value (PPV), and negative predictive value (NPV) were calculated. A two-sided *P* < 0.05 was considered statistically significant. The interobserver consistencies for all quantitative MRI parameters between the two radiologists were evaluated using the intraclass correlation coefficient (ICC) as follows: ICC ≥ 0.75 = strong; 0.4–0.75 = moderate, and ICC < 0.4 = weak.

## 3 Results

### 3.1 Inter-observer agreement of quantitative measurement

Interobserver agreement between the two experienced radiologists was strong for apparent diffusion coefficient (ADC) (ICC = 0.917), T1_pre_ (ICC = 0.863), T2_pre_ (ICC = 0.954), PD_pre_ (ICC = 0.975), T1_Gd_ (ICC = 0.889), T2_Gd_ (ICC = 0.934), and PD_Gd_ (ICC = 0.953).

### 3.2 Parameter values in differentiating benign and malignant breast lesions

Univariate regression analysis showed that age, lesion diameter, menopausal status, ADC, T2_pre_, PD_pre_, PD_Gd_, T2_Delta_, and T2_ratio_ were independent parameters for breast cancer diagnosis (*P* < 0.05). The ADC, T2_pre_, PD_pre_, PD_Gd_, T2_Delta_, and T2_ratio_ of malignant breast lesions were significantly lower than those of benign breast lesions. After excluding variables with a variance inflation factor (VIF) > 10 (T2_ratio_, T2_Delta_), multivariate analysis further showed that ADC and T2_pre_ were important independent factors for breast cancer diagnosis (*P* < 0.05) (**Table 3**).

### 3.3 Nomogram model building

**Figure 2:**
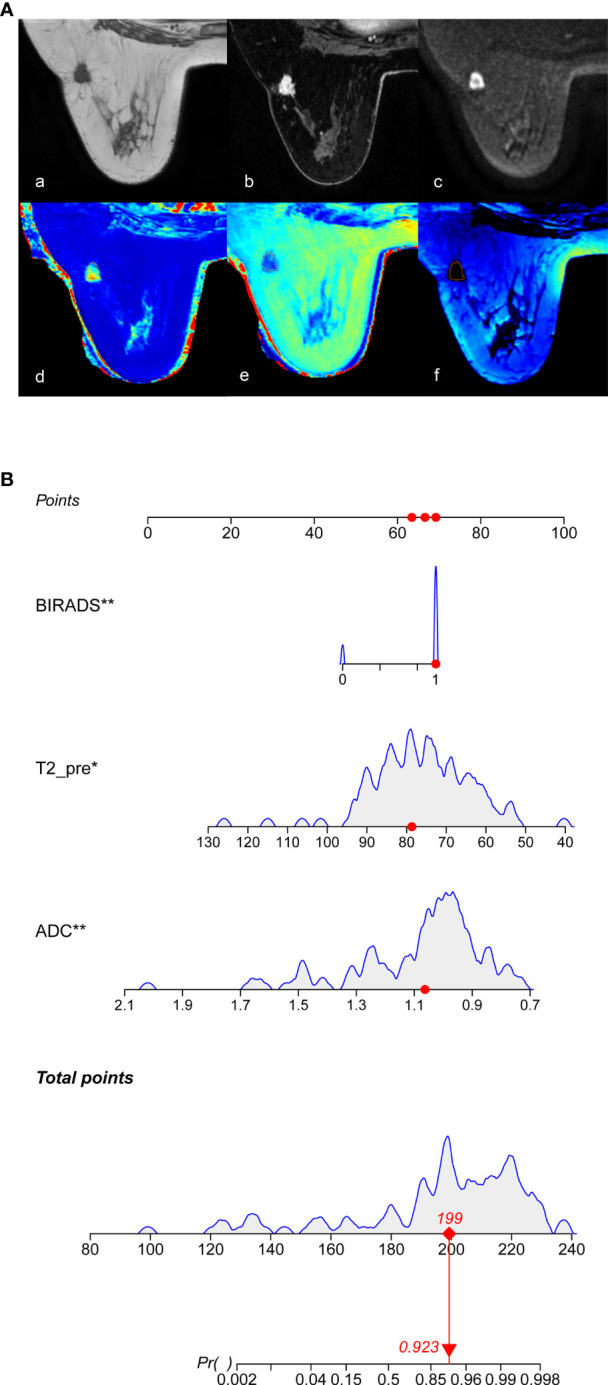
Nomogram for the diagnosis of benign and malignant breast lesions. **(A)** 37-year-old woman with a non-specific invasive ductal carcinoma, image with T2WI (A-a), DCE-MRI (A-b), MUSE-DWI (A-c), T1 map (A-d), T2 map (A-e), PD map (A-f). BI-RADS 4c=1 (i.e., malignancy could not be excluded, classified as malignant), T2_pre_ = 78.67 ms, ADC = 1.06×10^-3^ mm^2^/s. According to Nomograms **(B)**, total points was 199 points and the possibility of malignancy was about 92.30%.

**Figure 3:**
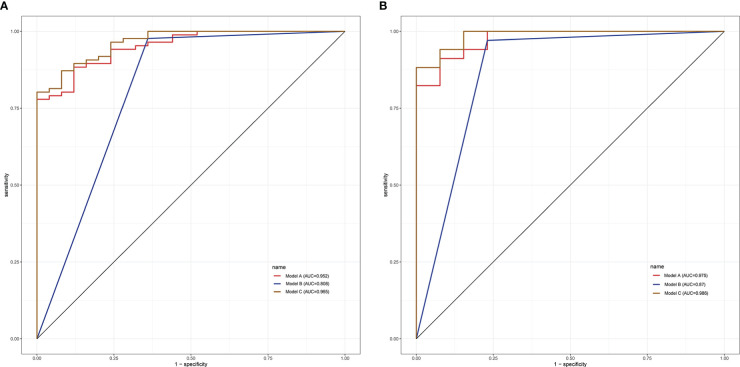
ROC curve analysis in the training group **(A)** and validation group **(B)**.

Based on the multivariate logistic regression model combined with DCE-MRI, the R statistical package was used to establish a nomogram for diagnosing benign and malignant breast lesions (**Figure 2**). The AUC of the nomogram model in the training and validation groups were 0.965 and 0.986, respectively (**Figure 3**). For example, in a 37-year-old woman with a non-specific invasive ductal carcinoma: T2WI (A-a), DCE-MRI (A-b), MUSE-DWI (A-c), T1 map (A-d), T2 map (A-e), PD map (A-f). BI-RADS 4c (i.e., malignancy could not be excluded, classified as malignant), T2_pre_ = 78.67 ms, ADC = 1.06×10^-3^ mm^2^/s. Total points = 199; probability of malignancy = 92.30% (**Figure 2**).

### 3.4 Value of prediction model in the identification of benign and malignant breast lesions

Based on the univariate and multivariate regression analyses, three prediction models were established: the quantitative parameter model (model A: ADC, T2_pre_), BI-RADS model (model B: BI-RADS), and multiparameter MRI model (model C: ADC, T2_pre_, BI-RADS).

In the training group, the AUCs of models A and C were 0.952 and 0.808, respectively, which were significantly higher than those of model B (Z = 2.94, P < 0.001; Z = -3.73, P < 0.001). The AUC of models A and C were not statistically significant (P = 0.288). In the validation group, model C had the highest diagnostic performance with an AUC of 0.975 (**Figure 3**, **Table 4**). As shown in **Table 4**, the model C had high efficacy in detecting malignancy, with sensitivity, specificity, accuracy, and PPV of 80.23%, 100.00%, 84.68%, and 100.00%, respectively, in the training group, and 97.06%, 92.31%, 95.74%, and 97.06% in the validation group, respectively. Model A had AUCs of 0.952 and 0.975 in the training group and validation group (95%CI:0.915-0.988, 0.939-1.000), indicating a better performance than model B(AUC:0.808,0.870; 95% CI:0.711-0.906,0.747-0.993). In the training group, model A had higher specificity and PPV compared to model B, and model B had improved predictive ability (accuracy, sensitivity, and NPV) compared to model A in distinguishing benign and malignant breast lesions (**Table 4**).

**Table 4:** Performance of the prediction model in the diagnosis of benign and malignant breast lesions in the training and validation groups.

Parameter	Training group			Validation group		
	Model A	Model B	Model C	Model A	Model B	Model C
AUC (95% CI)	0.952 (0.915-0.988)	0.808 (0.711-0.906)	0.965 (0.936-0.995)	0.975 (0.939-1.000)	0.870 (0.747-0.993)	0.986 (0.961-1.000)
Sen. (%)	77.91	97.67	80.23	91.18	97.06	97.06
Spe. (%)	100.00	64.00	100.00	92.31	76.92	92.31
Accuracy (%)	82.88	90.09	84.68	91.49	91.49	95.74
PPV (%)	100.00	90.32	100.00	96.88	91.67	97.06
NPV (%)	56.82	88.89	59.52	80.00	90.91	92.31

CI, confidence interval; Sen., sensitivity; Spe., specificity; PPV, positive predictive value; NPV, negative predictive value.

## 4 Discussion

This study investigated the diagnostic performance of a combination of syMRI, ADC, and BI-RADS in distinguishing benign and malignant breast lesions. The results revealed that quantitative syMRI parameters can be used as a reference index to identify benign and malignant breast lesions. Combined with BI-RADS, this can further improve the diagnostic efficiency of breast cancer.

Clinical DW imaging is based on SS-EPI, which is prone to image artifacts (24–28). Compared with sensitivity encoding alone, MUSE DWI has a better SNR, due to its improved matrix inversion conditioning, and has shown a high spatial resolution in previous investigations (29–31). However, few studies have been conducted on the diagnosis of breast diseases using MUSE sequences. Previous studies have focused on image quality comparison between MUSE-DWI and traditional SS-EPI sequences. Daimiel et al. showed that the ADC value in the MUSE sequence of breast cancer was significantly lower than that of benign breast lesions (11).

Similarly, this study found that the ADC values of malignant breast lesions were significantly lower than those of benign breast lesions. Owing to continuous cell proliferation, increased synthesis of macromolecular substances, such as proteins in the cytoplasm, and the release of many necrotic substances, the extracellular space reduces, content of bound water increases, and the diffusion of free water molecules is restricted. These factors decrease ADC values in breast cancer (18). Compared with the study by Daimiel et al., the number of patients included in this study was more prominent. Validation was performed in the validation group, indicating that the MUSE sequence has a massive advantage in benign and malignant breast lesions.

T1, T2, and PD are inherent properties of matter (16). Previous studies have shown that relaxation time is meaningful in the differential diagnosis of breast diseases, of which T2 relaxation time has a better diagnostic performance than other quantitative parameters (19, 20). Similarly, in this study, the T2 value of malignant breast lesions was significantly lower than that of benign lesions, indicating that the difference in T2 values is meaningful for distinguishing benign lesions from breast cancer. Previous studies have shown that the difference in relaxation time is related to the amount of free water (32–35). The higher the free water content, the longer is the relaxation time (18). In malignant lesions, continuous cell proliferation and the release of necrotic material lead to a reduction of the extracellular space. The above reasons may lead to the reduction of tissue-free water, which is the reason for the shorter T2 relaxation time in malignant lesions.

This study also investigated the quantitative parameters of post-enhanced syMRI; however, multivariate logistic regression analysis showed that post-enhanced MRI quantitative parameters (PD_Gd_) were not independent predictors of breast cancer. The reason behind this was analyzed because the post-enhanced syMRI sequence was performed after the enhanced sequence scan, and most of the contrast agent was cleared. Therefore, an enhanced quantitative relaxation index cannot represent the true blood supply to the tumor. This result was consistent with Matsuda et al. (21), suggesting that pre-enhanced MRI quantitative parameters were more meaningful. In this study, model A was established based on quantitative parameters (ADC, T2_pre_) and verified in the validation group. Compared with the conventional BI-RADS model(model B), model A showed better performance than model B. It shows that model A may be beneficial for pregnant women, children, and patients allergic to contrast media. In addition, the boundaries of small lesions are difficult to define, resulting in inaccurate measurements of relaxation values. Therefore, breast lesions smaller than 0.8 cm were excluded in this study, which was consistent with the exclusion criteria of Gao et al (20) and Matsuda et al (21). SyMRI can provide a variety of contrast images in a short scanning time, which is more helpful in obtaining histological information about lesions and improving the accuracy of benign and malignant breast identification.

DCE-MRI is an integral method for diagnosing breast diseases. However, its specificity varies greatly, which may result in clinically unnecessary biopsies. Therefore, effectively improving the diagnostic specificity of DCE-MRI is a future research direction for breast disease diagnosis. Different imaging parameters can provide different lesion characteristics. Therefore, this study combined different parameters to build a model and verified it in a validation group. This study showed that model A had better diagnostic performance (AUC = 0.952) than traditional BI-RADS model B (AUC = 0.808) in the training group. Model C had the highest diagnostic performance, with AUCs of 0.965 and 0.986 in the training and validation groups, respectively. In addition, an individualized prediction model for benign and malignant breast diagnosis was established based on the model C, which provided an intuitive and comprehensive prediction model for clinical practice to avoid unnecessary biopsies. In conclusion, the multiparameter MRI model has better diagnostic performance and provides objective quantitative parameters.

This study had some limitations. First, it was a single-center study with a relatively small sample size. Therefore, multicenter prospective studies are needed to verify the stability and reproducibility of different MRI scan parameters in the future. Second, when delineating the ROI, the most comprehensive axial level of the lesion was selected, and the entire lesion was not included, which may have resulted in loss of some heterogeneous features. Finally, this study adds MAGiC sequences before and after enhancement to conventional breast MRI, prolongs the examination time of patients, and reduces patient tolerance. It is necessary to optimize MAGiC scanning technology and shorten the examination time in future clinical practice.

## Data availability statement

The original contributions presented in the study are included in the article/supplementary material. Further inquiries can be directed to the corresponding author.

## Ethics statement

The studies involving human participants were reviewed and approved by General Hospital of Ningxia Medical University. Written informed consent for participation was not required for this study in accordance with the national legislation and the institutional requirements.

## Author contributions

JL: Investigation, Data curation, Writing-Original Draft, Writing-Review & Editing Preparation, statistical analysis; MX: Investigation, resources; JR: Writing - Review & Editing Preparation; ZL: Writing -Review & Editing Preparation; LX: resources, supervision; BC: Conceptualization, resources, supervision, project administration,writing - Review & Editing Preparation. All authors contributed tothe article and approved the submitted version.

## Glossary

**Table:**

ADC	Apparent diffusion coefficient
AUC	Area under the curve
BI-RADS	Breast Imaging Reporting and Data System
DCE-MRI	Dynamic contrast-enhanced magnetic resonance imaging
DWI	Diffusion weighted imaging
ICC	Intraclass correlation coefficient
MAGiC	MAGnetic resonance image Compilation
MRI	Magnetic resonance imaging
MUSE	Multiplexed sensitivity encoding
NPV	Negative predictive value
PD	Proton density
PD_Delta_	PD difference between before and after enhancement
PDGd	PD after enhancement
PD_pre_	PD before enhancement
PD_ratio_	PD ratios before and after enhancement
PPV	Positive predictive value
ROC	Receiver Operating Characteristic
ROIs	Region-of-interests
SNR	Signal-to-noise ratio
SS-EPI	Single-shot echo-planar imaging
syMRI	Synthetic magnetic resonance imaging
T1	longitudinal relaxation time
T1_Delta_	T1 difference between before and after enhancement
T1_Gd_	T1 after enhancement
T1_pre_	T1 before enhancement
T1_ratio_	T1 ratios before and after enhancement
T2	transverse relaxation time
T2_Delta_	T2 difference between before and after enhancement
T2_Gd_	T2 after enhancement
T2_pre_	T2 before enhancement
T2_ratio_	T2 ratios before and after enhancement
VIF	Variance inflation factor
